# Preoperative prealbumin and transferring: Relation to 30-day risk of complication in elective spine surgical patients: Erratum

**DOI:** 10.1097/MD.0000000000015153

**Published:** 2019-03-15

In the article, “Preoperative prealbumin and transferring: Relation to 30-day risk of complication in elective spine surgical patients”,^[1]^ which appears in Volume 98, Issue 9 of *Medicine*, the title should be “Preoperative prealbumin and transferrin: Relation to 30-day risk of complication in elective spine surgical patients.”
